# Brain NMDA Receptors in Schizophrenia and Depression

**DOI:** 10.3390/biom10060947

**Published:** 2020-06-23

**Authors:** Albert Adell

**Affiliations:** 1 Institute of Biomedicine and Biotechnology of Cantabria, IBBTEC (CSIC-University of Cantabria), Calle Albert Einstein 22 (PCTCAN), 39011 Santander, Spain; albert.adell@csic.es or albert.adell@unican.es; 2 Biomedical Research Networking Center for Mental Health (CIBERSAM), 39011 Santander, Spain

**Keywords:** NMDA, depression, schizophrenia, subunit, glutamate, GABA

## Abstract

N-methyl-D-aspartate (NMDA) receptor antagonists such as phencyclidine (PCP), dizocilpine (MK-801) and ketamine have long been considered a model of schizophrenia, both in animals and humans. However, ketamine has been recently approved for treatment-resistant depression, although with severe restrictions. Interestingly, the dosage in both conditions is similar, and positive symptoms of schizophrenia appear before antidepressant effects emerge. Here, we describe the temporal mechanisms implicated in schizophrenia-like and antidepressant-like effects of NMDA blockade in rats, and postulate that such effects may indicate that NMDA receptor antagonists induce similar mechanistic effects, and only the basal pre-drug state of the organism delimitates the overall outcome. Hence, blockade of NMDA receptors in depressive-like status can lead to amelioration or remission of symptoms, whereas healthy individuals develop psychotic symptoms and schizophrenia patients show an exacerbation of these symptoms after the administration of NMDA receptor antagonists.

## 1. Introduction

The N-Methyl-D-aspartate (NMDA) receptor (NMDAR) is an ionotropic glutamate receptor that possesses unique characteristics. The flow of ions through the channel is blocked by Mg^2+^. Two different processes are necessary for activating NMDARs. First, the previous membrane depolarization removes Mg^2+^ ions, and second, the additional binding of co-agonists glycine and glutamate allows voltage-dependent inflow of Na^+^ and Ca^2+^ ions and the outflow of K^+^ ions. This dual gating by ligand binding and membrane depolarization makes the NMDAR receptor optimally fitted to function as a coincidence detector [1]. NMDARs are involved in several physiologic functions, and their correct operation is crucial for cellular homeostasis. Any disruption in their function is thus susceptible of resulting in the manifestation of neuropsychiatric or neurological pathologies. NMDARs are critical for neuroplasticity, i.e., the ability of the brain to adapt to novel conditions. The function of NMDARs usually declines with age, which most likely contributes to the reduced plasticity that leads to learning and memory impairment. For this reason, the impairment of learning and memory seen in a variety of different pathologies, such as Alzheimer’s disease (AD), amyotrophic lateral sclerosis (ALS), Huntington’s disease, Parkinson’s disease (PD), schizophrenia and major depressive disorder (MDD) are associated with NMDAR malfunction. Due to the important implication of neuronal plasticity [2,3], the present review is focused on the link between NMDARs and the pathophysiology and treatment of schizophrenia and depression. Two of the most important mechanisms of synaptic plasticity that are dependent on NMDAR stimulation are long-term potentiation (LTP) and long-term depression (LTD). In LTP, a high-frequency stimulation of NMDARs produces a long-lasting increase in signal transmission between two neurons [4]. On the other hand, repetitive, low-frequency stimulation induces LTD by weakening specific synapses, which would counterbalance synaptic strengthening caused by LTP [5].

**Figure 1:**
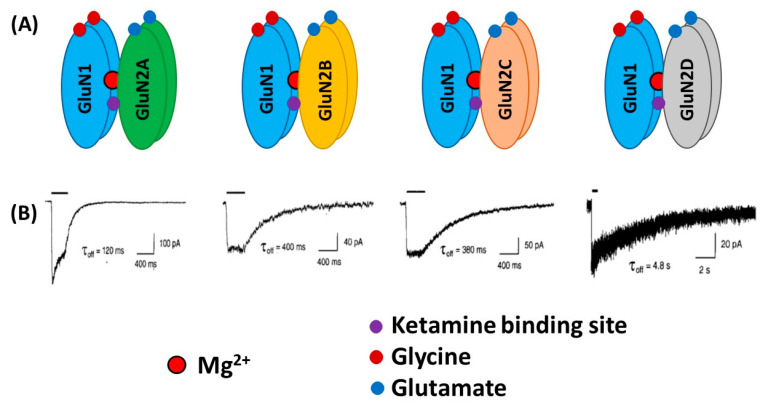
Schematic illustration of the N-Methyl-D-aspartate (NMDA) receptors (NMDARs) containing GluN1 and different GluN2 subtypes (**A**). Lower traces (**B**) indicate whole-cell patch-clamp recordings of responses from brief application of glutamate (1 ms of 1 mM glutamate) to recombinant diheteromeric NMDA receptor subtypes expressed in HEK293 cells. Averaged offset decay constant values (τ_off_) are listed below current traces. (**B**) “Reprinted from Neuron, Vol 12, number 3, H. Monyer, N Burnashev, D.J. Laurie, B. Sakmann, P.H. Seeburg, Developmental and regional expression in the rat brain and functional properties of four NMDA receptors, Pages No. 529-524, Copyright (1994), with permission from Elsevier”.

From a structural viewpoint, NMDARs are ionotropic glutamate receptors made up of four subunits. There are three different families of NMDAR subunits, i.e., GluN1, GluN2 and GluN3 (Figure 1). In addition, GluN2 subunits are subdivided into GluN2A, GluN2B, GluN2C and GluN2D subunits and GluN3 subunit into GluN3A and GluN3B subunits. The ion channel of the NMDAR is formed by two necessary GluN1 subunits, and either two GluN2 subunits or a combination of GluN2 and GluN3 subunits [6,7,8]. GluN1 subunits carry recognition sites for glycine, whereas GluN2 subunits possess recognition sites for glutamate, which determines the duration of channel opening and desensitization processes.

**Figure 2:**
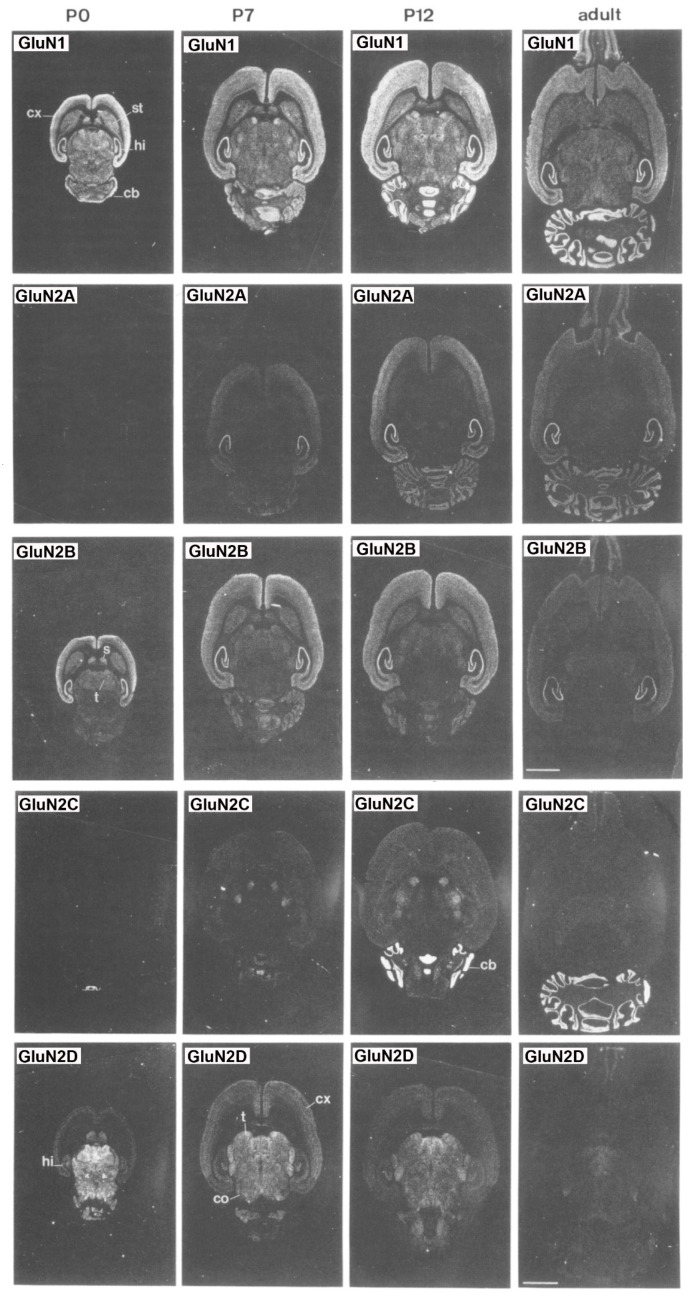
Distribution of the GluN1, GluN2A, GluN2B, GluN2C and GluN2D receptor subunit mRNAs. Postnatal developmental profiles of transcripts in horizontal rat brain sections from P0, P7, P12, and adult rats. Abbreviations: cb, cerebellum; cx, cortex; hi, hippocampus; s, septum; st, striatum; t, thalamus. Bar, 3.4 mm. “Reprinted from Neuron, Vol 12, number 3, H. Monyer, N Burnashev, D.J. Laurie, B. Sakmann, P.H. Seeburg, Developmental and regional expression in the rat brain and functional properties of four NMDA receptors, Pages No. 529-524, Copyright (1994), with permission from Elsevier”.

Overall, subunit composition of NMDARs changes along development and varies in different brain regions, which might influence the direction of synaptic plasticity. As depicted in Figure 2, the four glutamate-binding GluN2A-D subunits, in addition to the obligatory GluN1 subunit, are the most prominent subunits in the central nervous system (CNS) [9]. Cortical, hippocampal and striatal neurons in rodents are enriched in GluN2A and GluN2B subunits [8,10,11]. The GluN2D subunit is also present in the hippocampus, but only in younger rats, being undetectable in the adulthood [8]. In contrast, GluN2C subunits are practically restricted to cerebellum with low levels of expression in retrosplenial cortex and thalamus [8,12]. NMDARs are found mainly postsynaptically, although an important subset of them is also found extrasynaptically. The activation of synaptic NMDARs generally promotes synaptic and cell survival, whereas overactivation of extrasynaptic NMDARs by an excess of glutamate can be neurotoxic and induce cell death [13]. It has been reported that GluN2A subunits are predominant at the synapses, whereas GluN2B and GluN2D are localized, though not exclusively, to extrasynaptic compartment [14,15,16,17]. Thus, GluN2A-containing receptors have been reported to contribute to synaptic plasticity, whereas GluN2B-selective antagonists may possess neuroprotective properties.

Over the last few decades, a plethora of articles have reported findings of the ability of targeting NMDARs for treating different pathologies. However, most clinical trials have failed to show significant efficacy, while exhibiting adverse effects [18,19].

## 2. NMDA Receptors in Schizophrenia

### 2.1. Clinical Evidence

The first clue of the implication of NMDARs in schizophrenia resulted from the observations that NMDAR blockers, such as phencyclidine (PCP) and ketamine, induced in healthy individuals psychotic and negative symptoms, as well as cognitive impairment, that resemble those present in schizophrenia [20,21,22,23] and exacerbated these symptoms in schizophrenic patients [24,25]. In addition, ketamine also induced a reduction of NMDA receptors in the human brain, which strongly correlated with negative symptoms [26]. More recently, neuroimaging studies have shown, for the first time, direct in vivo evidence of a reduction of NMDA receptors in the left hippocampus of medication-free schizophrenic patients [27]. Schizophrenic patients also exhibit deficits in the attention and information (cognitive and sensorial) processing measured through the prepulse inhibition (PPI) of the acoustic startle response [28,29,30]. PPI is known to prevent the organism from receiving an overload of information, a behavioral event altered in schizophrenia, which is reflective of abnormal functioning of the corticostriato-thalamocortical circuitry. Therefore, thalamic gating deficits would result in an excessive transfer of information to cortical structures and the subsequent cognitive deficiency. However, reductions in PPI were not observed in healthy individuals after the administration of ketamine [31], which is in sharp contrast to the effects consistently seen in rodents (see below). Another electrophysiological operation impaired in schizophrenia is mismatch negativity (MMN). MMN is an auditory event-related response in an electroencephalographic (EEG) signal, which occurs when a sequence of repetitive sounds is interrupted by an occasional “oddball” sound that differs in frequency (pitch) or duration. This sensory (auditory) information processing is also damaged, not only in schizophrenic patients [32], but also in their relatives [33,34], and it is reported to represent error in prediction. Interestingly, healthy individuals exhibit MMN after a single dose of ketamine [35]. Taking all these findings together, it is evident that not all components of the symptoms of schizophrenia in human beings are caused by a direct hypofunction of NMDARs. Hence, it appears that MMN is dependent on NMDAR blockade, whereas PPI is not. Further research is needed to understand why these actions are species specific.

#### 2.1.1. Neurophysiology

Brain oscillations have been also studied as possible in vivo biomarkers of the illness. Schizophrenic patients frequently show EEG abnormalities [36,37] and a closer look of these changes can bring some evidence about the pathogenesis of the illness. Oscillations in the γ band (30–100 Hz) have been the object of interest, because of their involvement in cognitive functions known to be impaired in schizophrenia [37,38,39,40,41,42,43]. Cortical γ oscillations result from the control that parvalbumin-containing γ-aminobutyric acid (GABA)ergic interneurons exert over pyramidal neurons [44,45] and, in this regard, abnormalities in these cortical interneurons have been consistently found in schizophrenia [42,46,47,48]. A preferential blockade of NMDA receptors on parvalbumin-expressing (PV) interneurons is postulated as a central mechanism of classical actions of NMDA receptor antagonists, which results in increased cortical activity and γ-band oscillations [49,50,51,52,53]. Further, schizophrenia is characterized by abnormalities in γ oscillations measured in different cognitive tasks. Thus, schizophrenic patients exhibit increases in spontaneous gamma band power [42,43], which have been related to positive symptoms [54,55,56]. However, marked deficits in the γ frequency band were observed when γ oscillations were examined under different tasks (for instance, auditory-evoked responses) [39,42,57,58]. However, at variance to what happen in rodents (see below), NMDAR antagonists, such as ketamine, usually elicit increases in spontaneous γ band power, probably evoked by an excessive stimulation of glutamatergic transmission in cortical and subcortical areas [42,43,59,60,61]. Although no direct measure of brain glutamate level can be determined from the brain of schizophrenics, some indirect estimates have implicated increased activation of prefrontal cortex in first-episode schizophrenia [62,63,64] and after ketamine administration [65,66], which can be taken as suggestive of increased glutamate release in the human brain.

In addition to high frequency oscillations, low frequency oscillations (α, θ and δ bands) are also related to cognitive processing. For instance, the α wave range (8–12 Hz) is related to working memory processes [67], whereas the θ range (5–8 Hz) is involved in attention and signal detection [68], which reflects cortico-hippocampal interactions [69,70,71]. On the other hand, oscillations in the δ band are also involved in decision making procedures [68].

#### 2.1.2. Post-Mortem Studies

Another important line of investigation aimed at examining the implication of NMDAR in schizophrenia has been the study of post-mortem tissue. Thus, decreased cortical expression of NMDAR subunits have been observed in subjects with schizophrenia, though not in a consistent manner [72], depending on the brain region examined and methodology used. In this regard, decreased transcripts coding for the GluN1 subunit have been found in the prefrontal cortex [73,74] and hippocampal subregions [75,76]. However, as aforementioned, care must be taken, because some of these changes are also observed in different psychiatric conditions and, by no means, are compelling, as long as contradictory results have been found. As a matter of fact, this same meta-analysis found no consistent statistically significant changes in cortical mRNA and protein expression of GluN2A, GluN2B and GluN2D subunits in schizophrenia, with the exception of decreased expression of mRNA coding for GluN2C [74,77,78]. Of note, in the postsynaptic density compartment of human post-mortem prefrontal cortex, an important reduction in the density of the postsynaptic protein PSD-95 [74] and in the activity of signaling cascades downstream of the NMDAR has been found in schizophrenia [79].

Since the finding that the therapeutic efficacy of antipsychotic drugs was directly correlated to their affinity for dopamine D2 receptors [80,81], it was first postulated that altered dopamine D2-like receptors was responsible for schizophrenia symptoms. However, this was not confirmed until the study by Abi-Dargham [82], which reported increased occupancy of dopamine D2 receptors in schizophrenia. Moreover, it was evidenced that the administration of ketamine to healthy subjects enhanced the release of dopamine in ventral striatum, which was shown to correlate strongly with the emergence of psychotic symptoms [83]. For these reasons, an association between D2-like receptors and NMDA hypofunction was hypothesized [84,85]. In addition to these findings, the differential expression of some splice variants might also evoke abnormal NMDAR trafficking and plasma membrane insertion, which may lead to abnormalities seen in schizophrenia patients [86,87].

Another important issue is the role of astrocytes in schizophrenia. High levels of extracellular glutamate are associated with excitotoxicity, and astrocytes are the principal cells that contribute to glutamate homeostasis through its removal from the synapsis, by means of selective reuptake mechanisms. The excitatory amino acid transporters 1 and 2 (EAAT1/2) are predominantly localized on astrocytes and are mainly responsible for clearing synaptic glutamate and influencing postsynaptic responses. Post-mortem studies have reported that EAAT1 expression is decreased in schizophrenia, as compared to healthy subjects [88]. Additionally, the activation of EAAT2 and its transport to plasma membrane was found to be reduced in schizophrenic brains [89]. A genetic variant of EAAT2 (SNP rs4354668) has been correlated with the severity of schizophrenia [90]. Altogether, these findings are consistent with an impairment of astrocyte function in schizophrenia [91].

### 2.2. Preclinical Evidence

**Figure 3:**
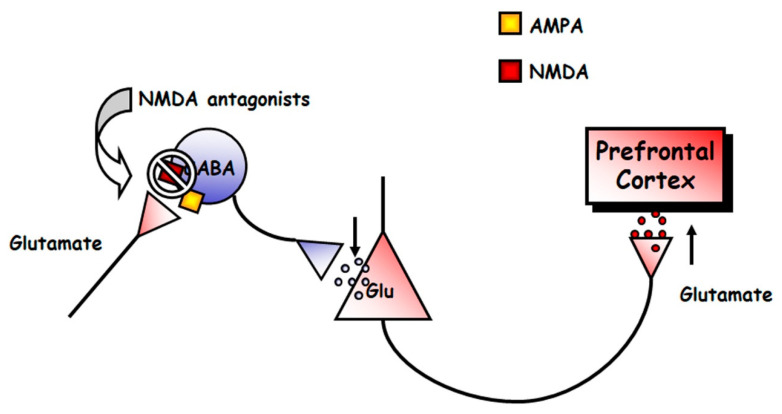
Scheme of the mechanism of action of NMDAR antagonists. These drugs would block NMDAR in a population of tonically active γ-aminobutyric acid (GABA)ergic neurons. This would decrease the activity of these neurons, and the consequent decrease in GABA release would cause the disinhibition of glutamatergic neurotransmission. The glutamate released would lead to the stimulation of α-amino-3-hydroxy-5-methyl-4-isoxazolepropionic acid (AMPA) receptors in pyramidal cells, which would result in the described state of hyperactivity.

Mental disorders, such as schizophrenia and depression, are illnesses uniquely human, in that they are diagnosed using different interview questionnaires. Therefore, no animal model can recreate the full spectrum of their symptoms. However, some models exist that examine changes in behavioral and neurophysiological readouts that are compatible with changes that are also observed in patients. For instance, the acute, systemic administration of NMDAR antagonists induces, in rodents, hyperlocomotion and stereotypical behaviors [92], which are potentially compatible to positive symptoms of schizophrenia [93,94], in that they are associated with increased dopaminergic and serotonergic transmission in the brain [95,96]. NMDAR antagonists, such as PCP and dizocilpine (MK-801), also produce severe disruptions in PPI, and deficits in different domains of cognition in rats [97,98]. Acute NMDAR antagonism also evokes an increased firing rate of pyramidal neurons and expression of c-fos mRNA of the prefrontal cortex [99,100,101,102,103], which suggests an overall, excessive prefrontal activity, which results in elevated release of glutamate [104,105,106], dopamine [107,108,109], 5-HT [110,111,112] and acetylcholine [113,114] in the medial prefrontal cortex (mPFC) of rats. Altogether these findings indicate that an overstimulation of different transmitter systems in the mPFC is a general response to NMDAR hypofunction, which can account for the behavioral effects induced by NMDAR antagonists. The enhanced release of monoamines most likely result from the stimulation of prefrontal excitatory glutamatergic inputs onto midbrain dopamine and 5-HT cell groups, as suggested by recent investigations [115,116]. It is paradoxical that a blockade of excitatory glutamatergic receptors, such as NMDARs, results in the stimulation of glutamate release. The most accepted hypothesis to explain this phenomenon is the disinhibition theory [49,104,117], which postulates that NMDA antagonists would block NMDA receptors located on tonically active GABAergic neurons, which would control glutamatergic output. This would diminish GABAergic inhibition, thus disinhibiting glutamatergic neurotransmission impinging upon α-amino-3-hydroxy-5-methyl-4-isoxazolepropionic acid (AMPA) receptors (Figure 3). The finding that some GABAergic interneurons in hippocampus and neocortex are enriched in NMDAR and receive a greater glutamatergic input in comparison with pyramidal neurons, gives further cellular support to this view [118,119].

Interestingly, it has been reported that an action of NMDAR antagonists on the mPFC of both brain hemispheres is needed to model these changes in the mPFC [102]. In addition, transmitter changes have been found in other brain areas. For instance, noncompetitive NMDAR antagonists also enhance the efflux of glutamate in the nucleus accumbens [120], acetylcholine in the retrosplenial cortex and hippocampus [121,122], 5-HT in the nucleus accumbens [111], noradrenaline in nucleus accumbens and hippocampus [123,124,125] and dopamine in limbic areas, such as the nucleus accumbens, hippocampus and ventral pallidum [108,111,113,126,127,128], although these effects were less pronounced than in the mPFC.

#### 2.2.1. Neurophysiology

Abnormal oscillatory patterns have been also observed in rodent models. Hence, systemic administration, and even the local administration of PCP, MK-801 or ketamine into some brain regions, increase γ and high frequency oscillations (HFO) in a number of cortical and subcortical structures (see [129], for a review). It has also been observed that ketamine and MK-801 reduced the frequency and power of θ oscillations in the hippocampus [130,131]. Furthermore, acute PCP also alters thalamo-cortical oscillations, particularly those below the 4 Hz band [132].

#### 2.2.2. Animal Models

The modeling of schizophrenia has been achieved not only by acute, but also by long-term administration of NMDAR antagonists [133]. Thus, although the nature of some changes is similar, following acute or protracted treatment with NMDAR antagonists, it has been postulated that changes after the acute regimen are more comparable with those occurring in early stages of schizophrenia, whereas the duration of such changes after sustained administration appears to be more related to the persistence of clinical symptoms of the illness [134,135,136,137,138]. Acute PCP treatment increased locomotor activity in rodents, an effect potentiated after long-term treatment [139,140,141]. Subchronic PCP treatment does not seem to affect basal and PCP-induced 5-HT efflux in the mPFC. However, in comparison to acute administration, subchronic PCP attenuated basal prefrontal dopamine release, but potentiated PCP-induced dopamine efflux. The reduced basal extracellular concentration of dopamine could be accounted for by lowering its synthesis, as measured by a diminished expression of tyrosine hydroxylase mRNA in the ventral tegmental area [141].

Preclinical evidence from animal models also reported impairment of astrocyte function in the schizophrenia model of repeated MK-801 exposure [142]. In addition, abnormal EAAT1/2 function is associated with schizophrenia phenotypes. For instance, mice lacking EAAT1 showed hyperlocomotion and increased sensitivity to the locomotor hyperactivity produced by NMDAR antagonists [143]. In addition, the hyperlocomotion of these EAAT1 knockout mice was reversed by the antipsychotic haloperidol.

## 3. NMDA Receptors as Target for Treatment in Schizophrenia

A plethora of antipsychotic drugs have been approved for the treatment of schizophrenia, most of them targeting monoamine receptors. Nevertheless, although hypofunction of NMDA neurotransmission has been shown to play an important role in the pathophysiology of schizophrenia, the results of the clinical trials of the NMDA-enhancing agents have been inconsistent. Since direct agonists of the NMDA receptor can produce severe excitotoxic effects, the therapeutic focus turned to the obligatory glycine co-agonist site of the NMDAR (NMDA-glycine site). Thus, drugs stimulating the NMDA-glycine site were postulated to be effective in treating the symptoms of schizophrenia [144]. These types of drugs include glycine itself [145,146,147], other agonists of the NMDA-glycine recognition site such as D-serine [148,149,150] and the partial agonist D-cycloserine [151,152,153,154,155], as well as inhibitors of glycine transporters, such as sarcosine (*N*-methyl-glycine), which increase the synaptic availability of glycine [156,157]. However, although preclinical studies in rodents showed that partial glycine site agonists and glycine reuptake inhibitors exhibit comparable pro-cognitive effects with the potential for the treatment of schizophrenia [158], a double-blind, randomized clinical trial concluded that neither glycine nor D-cycloserine is a generally effective therapeutic option for treating negative symptoms or cognitive impairments [147]. As a matter of fact, most clinical trials conducted to date have failed to show efficacy of these agents for the treatment of schizophrenia [159].

First generation (typical) antipsychotics like haloperidol and chlorpromazine potently block dopamine D_2_/D_3_/D_4_. The blockade of dopamine D_2_ receptors in the mPFC is postulated to alleviate psychotic symptoms (delusions, hallucinations). However, the same action in other areas of the brain can cause severe extrapyramidal side-effects (EPS) and hyperprolactinemia [160]. Second generation (atypical) antipsychotic drugs, like clozapine and olanzapine, retain some degree of dopamine D_2_/D_3_/D_4_ antagonism, but they possess a superior antagonism at 5-HT_2A/2C_ receptors. These features seem to be more effective for negative symptoms and cognitive deficits, although some side-effects (weight gain, impairment of glucose and lipid metabolism) may emerge [161]. Microdialysis studies carried out in our lab showed that the NMDAR antagonist, MK-801, elevated the release of glutamate and 5-HT in the mPFC [162,163]. Our results showed that typical antipsychotics, such as haloperidol and chlorpromazine, and atypical antipsychotics, such as clozapine and olanzapine, were able to attenuate the excess of prefrontal glutamate, but only atypical drugs were able to further reduce the excess of prefrontal 5-HT [163]. Thus, our results further suggest that the blockade of an exacerbated 5-HT release in the mPFC induced by NMDAR antagonists can be a good indicator of “atypicality” of antipsychotic drugs. Although this has been established for clozapine and olanzapine (drugs that display a similar pharmacological profile), further research is needed to determine whether this is a distinct feature of other antipsychotic drugs. Interestingly, these effects of haloperidol and chlorpromazine could be mimicked by dopamine D_2_/D_3_/D_4_ receptor antagonists, and those of clozapine and olanzapine were reproduced by 5-HT_2A_ receptor antagonists and 5-HT_1A_ receptor agonists [162,163]. Therefore, it seems that dopamine D_2_/D_3_/D_4_ receptor antagonism is relevant to treat positive symptoms of schizophrenia, but it is conceivable that 5-HT_2A_ receptor antagonism and 5-HT_1A_ receptor agonism, may contribute to a better profile of antipsychotic treatment. In fact, preclinical studies have shown that 5-HT_2A_ antagonists and 5-HT_1A_ agonists can alleviate the cognitive deficits induced by NMDA receptor antagonists [164]. Each of these receptor components do not confer antipsychotic properties individually, so it is likely that a combined effect is needed.

## 4. NMDA Receptors in Depression

### 4.1. Clinical Evidence

Four different findings suggest a relationship between dysfunction of NMDAR and depression. First, an anomalous gene expression of NMDAR has been found in depressed people [165,166,167]; second, stressors induce excessive NMDAR activity that could result in the pathology of depression [168]; third, NMDAR blockers, such as ketamine (see below), have antidepressant properties [169,170,171]; and fourth, conventional antidepressant drugs usually impact on NMDAR function [172,173,174]. Altogether, these investigations suggest an overstimulation of NMDAR in major depression [175,176,177,178].

#### Post-Mortem Studies

However, the results obtained from these investigations exhibit great variability, and are far from consistent, likely due to differences in the brain structure examined or the methodological procedures used. For instance, post-mortem studies have revealed reduced levels of GluN2A and GluN2B subunits in the prefrontal [179] and perirhinal [180] cortices, but increased levels of GluN2A subunits in the lateral amygdala [181] in major depression. Previous post-mortem work did not find changes in the total content of GluN1 protein in the prefrontal cortex in depression [179,182]. Yet, when splice isoforms were considered, NMDAR activity and the GluN1 subunit carrying the C1 cytosolic segment were found to be increased in depressives [182]. Another report also described that the GluN2C subunit is elevated in the locus coeruleus of patients with major depression [183]. Further, early life adverse effects reduce NMDAR binding in dorsolateral prefrontal and anterior cingulate cortices, which could result from excessive NMDAR stimulation [184]. This would be consistent with the hypothesis of glutamate excitotoxicity produced by stress-induced excessive NMDAR activity, which could induce depressive states [168,185].

Further work has reported higher expression levels of the NMDAR subunit genes, GRIN2B and GRIN2C, in the locus coeruleus of depressed patients [186]. Another study reported a higher expression of GRIN1, GRIN2A, GRIN2B, GRIN2C and GRIN2D subunit mRNAs, but only in female MDD patients. Nevertheless, when male and female patients were grouped, the expression of GRIN2B mRNA was higher in those who committed suicide, in comparison with those that suffered depression but did not die by suicide [187]. For this reason, GRIN2B mRNA level is rather considered as biomarker of suicide and, in fact, polymorphisms of GRIN2B have been postulated to predict treatment-resistant depression [165,188]. Further epigenetic work showed that methylation in GRIN1 was a significant predictor of depression in a sample of maltreated children [189].

Although there is a great number of genome wide association studies (GWAS) that have examined genetic changes in depression, much less attention so far has been devoted to the study of gene methylation. In this regard, only one study has found a hypermethylation of the GRIN2A gene body in the hippocampus and prefrontal cortex of post-mortem human tissue in depression [167], which has been attributed to overexpression of the GluN2A subunit [190].

Other post-mortem studies have revealed the influence of astrocytes in MDD (see [191,192] for review). Thus, there is mounting evidence that the number and morphology of astrocytes are altered in depression, particularly in the frontal cortex [193,194,195] and the dentate gyrus of the hippocampus [196]. A significant reduction of the packing density of glial fibrillary acidic protein (GFAP)-containing astrocytes was also found, but only in younger (30–45 years old) patients [197].

### 4.2. Preclinical Evidence

There is substantial evidence from rodent models relevant to depression that stress induces glutamatergic hyperactivity, as well as the overexpression of NMDARs [198,199,200]. Given that NMDAR antagonists exert a preferential blockade of NMDAR on PV interneurons, enhanced PV interneuron activity has been observed after stress and might underlie depression-like behavior [201,202,203]. However, this is not a universal picture inasmuch as decreased PV cell activity has been observed after different stressful conditions (see [204], for review).

Maternal separation induces increased expression of GluN2A (but not GluN2B) subunit of NMDARs in the hippocampus of adult rats [205]. Chronic restraint stress significantly elevated GRIN2a (GluN2A) and GRIN2b (GluN2B) subunit genes in BALB/c mice, but not in C57BL/6 mice [206]. Chronic restraint stress also increased the levels of GRIN1 mRNA, along with a reduction in protein levels in dorsal hippocampus [207]. Chronic corticosterone administration, which emulates the endocrine response to stress, increased GRIN2A and GRIN2B mRNAs, which mediated the deleterious effects on the hippocampus [208]. Further, the olfactory bulbectomy model of depression reduces NMDA receptor binding in the prefrontal cortex and amygdala [209,210]. On the other hand, in the frontal cortex, BDNF deficiency, which occurs under chronic stress and is one of the leading causes of depression, also increased the density of GRIN1, GRIN2A and GRIN2B genes in the early stages of development [211]. Therefore, with all these findings taken together, a logical reasoning would hypothesize that the deletion or inhibition of NMDAR subunits would have antidepressant-like effects. Indeed, inactivation of the GluN2A subunit has been shown to evoke antidepressant-like activity in mice [212]. Yet, the deletion of the GluN2D subunit in the bed nucleus of the stria terminalis (BNST) increases depressive-like behaviors [213]. However, mice with constitutive, global deletion of the GluN1 or GluN2B subunits die neonatally. Homozygous GRIN1 knockout mice only survive 8–15 h after birth [214], and homozygous GRIN2b knockout mice die at early postnatal stages, because of an impaired suckling response [215].

However, the same as occurs with human studies, work with experimental animals has also yielded contradictory results. Thus, increased GRIN1 mRNA expression in the mPFC appears to have antidepressant-like effects in the forced swim test. [216]. GRIN1 was also found to be downregulated in chronic unpredictable mild stress (CUMS) [217]. The type and duration of the stressor, as well as the brain region examined, may underlie these differences.

Alterations in the number and/or function of astrocytes have also been found in animal models of depression (see [218] for review). Indeed, a reduced number and volume of astrocytes was found in the prefrontal cortex [219] and the hippocampus [220], in the chronic unpredictable stress procedure and chronic social defeat stress paradigm. Interestingly, neurotoxic lesioning of astrocytes in the prefrontal cortex is sufficient to induce depressive-like behaviors in rodents [221], an effect also found after knocking-down the expression of astrocytic glutamate transporter GLAST/GLT-1 in the prefrontal cortex of the mouse, using small interfering RNA (siRNA) strategies [222].

## 5. NMDA Receptors as Target for Treatment in Depression

**Figure 4:**
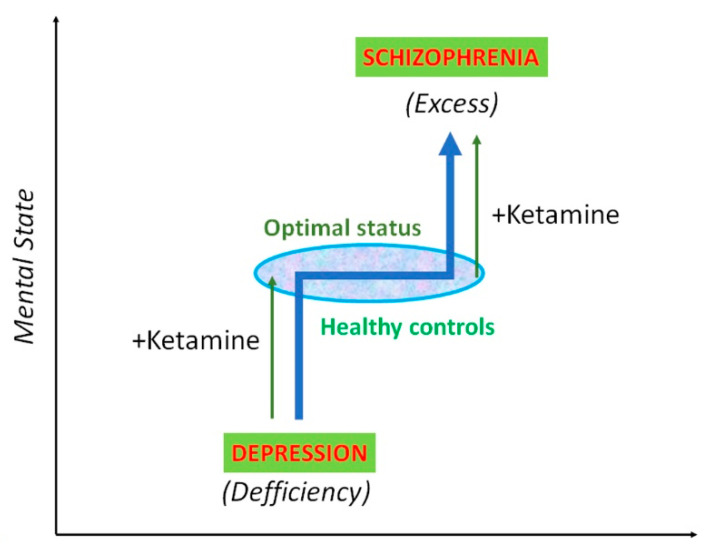
Schematic representation of mental state that appears as a continuum that can be modulated by ketamine. Ketamine can alleviate depressive symptoms in patients, but also induce psychotic symptoms when administered to healthy subjects. The terms deficiency and excess are abstract concepts herein, but can refer, for instance, to cortical monoamine levels.

Conventional antidepressant drugs inhibit monoamine transporters, based upon the assumption that a deficit in the synaptic concentration of monoamines are the underlying cause of depression. However, monoamine-based antidepressants have important limitations, such as lower efficacy, therapeutic delay and, above all, the existence of a population of patients (estimated as one third of depressed people) that do not respond to the treatment [223,224,225]. Thus, alternative therapeutic goals have focused on the neurotransmitter glutamate and its receptors, particularly in the ionotropic NMDAR. The first indication that NMDAR blockers had antidepressant-like effects dates back to 1990, when Trullas and Skolnick [226] reported that both competitive and non-competitive NMDAR antagonists reduced immobility in the forced swim (FST) and tail suspension (TST) tests. Three decades later, and after intensive research, esketamine (Spravato^®^) was approved by the US Food and Drug Administration (FDA) and the European Medicines Agency (EMA) for adults with major depression who are resistant to treatment, although with rigorous restrictions. In the past decade, clinical investigations have shown that a single intravenous bolus administration of the non-competitive NMDAR antagonist, ketamine, evoked a rapid (in only 2 h) and sustained (lasting up to 7 days) antidepressant action [169,170,227,228]. Interestingly, the dosage of ketamine used for antidepressant action (a total dose of 0.5 mg/kg infused over 40 min) is comparable to the one used to evoke psychotic symptoms in healthy volunteers [21]. Thus, it seems that ketamine can exert a similar enhancing modulatory function in mental status, i.e., increasing the emotional condition and mood in depressives, and increasing extremely disordered thinking and behavior in healthy controls, and both conditions can be triggered by stress (Figure 4). Although the mechanism of action of ketamine is not completely understood, the involvement of several cellular and molecular processes has been unveiled. Hence, its antidepressant-like action requires the activation of another class of ionotropic glutamate receptors, the α-amino-3-hydroxy-5-methyl-4-isoxazolepropionic acid (AMPA) receptors [229,230,231], and the stimulation of mammalian (or mechanistic) target of rapamycin (mTOR), an intracellular pathway associated with synaptic plasticity [232,233]. Further, it has been observed that ketamine also facilitates the expression of the GluA1 subunit of the AMPA receptor [232,234,235,236]. mTOR seems to be specific to rapid-acting antidepressants, because other conventional antidepressant drugs, such as imipramine and fluoxetine, do not require mTOR signaling. mTOR is a serine/threonine protein kinase that regulates the initiation of protein translation, thus inducing the protein synthesis required for synaptogenesis [237,238], a process needed for antidepressant action. As a matter of fact, recent studies have found reduced mTOR function in the prefrontal cortex of depressives [239], and in the frontal cortex, amygdala and hippocampus of rats exposed to chronic stress [240,241,242].

**Figure 5:**
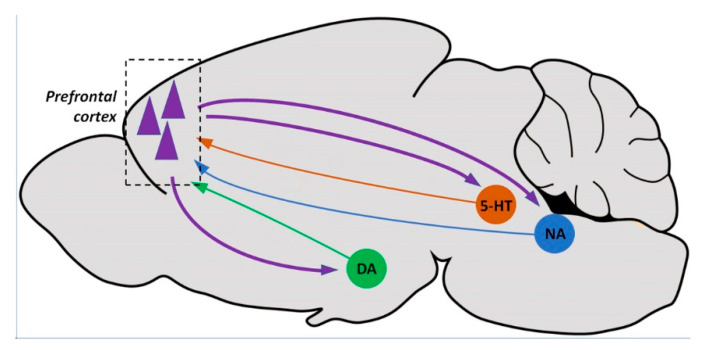
Prefrontal pyramidal neurons (dashed line square) project to the dopaminergic neurons of the ventral tegmental area, serotonergic neurons of the dorsal raphe nucleus and noradrenergic neurons of the locus coeruleus. Neurons from these monoaminergic nuclei send projections back that modulate the neuronal activity of prefrontal cortex.

The antidepressant-like actions of systemic ketamine were reproduced by the microinfusion of the drug into the infralimbic cortex [243,244], thus underscoring the importance of this brain region in the antidepressant effects of ketamine. Furthermore, the optogenetic stimulation of the pyramidal cells in the mPFC that project to the dorsal raphe nucleus (DRN, the nucleus where most serotonergic neurons that innervate forebrain structures originate) produces antidepressant effects [243,245,246,247], which reproduced the response elicited by the intracortical administration of ketamine [243]. Therefore, it is postulated that the rapid antidepressant-like effects of ketamine are accounted for by the glutamate-induced AMPA receptors localized to layer 5 pyramidal neurons that project to the DRN [243], thus releasing 5-HT in the mPFC, which would contribute to the antidepressant response. Given that ketamine also elevated the release of dopamine and noradrenaline in the mPFC, it is conceivable that ketamine would also activate the projections from the mPFC to the dopaminergic ventral tegmental area and the noradrenergic locus coeruleus. Altogether, the rapid enhancement of the release of 5-HT, dopamine and noradrenaline in the mPFC would contribute to the rapid antidepressant response of ketamine [116] (Figure 5).

Indeed, the mPFC has been traditionally implicated in alertness and attentional processes, working memory and behavioral flexibility, behaviors known to be associated with noradrenaline, dopamine and 5-HT, respectively, and impaired in mood disorders. However, despite the widespread recognition of the involvement of monoamines in depression [248], there is a lack of compelling evidence linking depression to low serotonergic and/or noradrenergic and/or dopaminergic transmissions. A possible explanation could be that a greater than 90% depletion of one of more monoamines would be required for the emergence of depressive states. For instance, serotonin depletion does not usually alter immobility in the FST [249], but we recently demonstrated that, when this depletion was 90%, immobility was significantly increased [115]. Severity of depression is positively associated with the frequency and severity of somatic symptoms [250,251,252,253], which depends on the monoamine levels [254,255]. Old clinical work, which would be hardly reproducible in present days for obvious bioethical reasons, showed that, in hospitalized, depressed patients who had responded to treatment with tranylcypromine (a monoamine oxidase inhibitor) or imipramine (a tricyclic antidepressant), depressive symptomatology returned after small doses of parachlorophenylalanine (an irreversible inhibitor of the synthesis of 5-HT) [256,257]. Perhaps this has been the only work so far that evidenced the appearance of depression symptoms after the depletion of body 5-HT.

As aforementioned, the approval of the (*S*)-isomer of ketamine has come with serious restrictions, because of the drug can cause dissociation and delirium at the low doses used for depression. These adverse effects appear shortly after infusion onset but vanish just before the antidepressant response begins [169]. In attempts to overcome such problems, other ketamine-based alternatives have been pursued. The approved (*S*)-ketamine has ~4-fold greater affinity for NMDARs than the (*R*)-ketamine. On the other hand, (*S*)-ketamine displays a greater anesthetic potency and greater undesirable psychotomimetic side effects than (*R*)-ketamine [258,259]. Furthermore, a recent study by Hashimoto and co-workers established that (*R*)-ketamine had greater potency and longer-lasting antidepressant effects than (*S*)-ketamine in a rodent model of depression [260]. For this reason, this group of investigators proposed the R-stereoisomer of ketamine as an alternative for treatment-resistant major depression [261], because (*R*)-ketamine does not seem to cause psychotomimetic behaviors, neurotoxicity and abuse potential in animal models [260,262,263]. To the best of my knowledge, at present, there is only one open-label pilot study published that examined the antidepressant effects of (*R*)-ketamine [264] and one phase I clinical trial that evaluates the safety and pharmacokinetics of (*R*)-ketamine in healthy subjects (ClinicalTrials.gov Identifier: NCT04108234).

Another strategy has been to examine the antidepressant effects of subunit-selective NMDARs. Ketamine is a non-subunit-selective NMDAR antagonist, and it was hypothesized that perhaps other NMDAR antagonists selective for GluN2A or GluN2B would possess antidepressant activity in the absence of psychotomimetic effects. In this regard, preclinical studies have shown that the GluN2B subunit NMDAR receptor antagonist, Ro 25-6981, possesses antidepressant-like effects [229,232]. Furthermore, in the clinic, several investigational drugs have exhibited some efficacy in the treatment of depressive states, such as the GluN2B receptor antagonists CP-101,606 [265]) and MK 0657 [266], the NMDAR glycine site partial agonist rapastinel (GLYX-13) [267], and the low-trapping nonselective NMDA channel blocker lanicemine (AZD6765) [268,269]. However, the rapid and robust effects of ketamine are clear, whereas the effects of MK 0657 and lanicemine are comparatively modest and short-lived. Although these compounds showed an initial promise for treating depression, further clinical studies failed to exhibit efficacy and the research and development of these compounds were eventually discontinued. In contrast, less attention has been paid to GluN2A antagonists. To this end, we assessed the biochemical and behavioral changes elicited by NVP-AAM077 [270], a competitive antagonist showing ~10-fold greater selectivity for the rat GluN2A than for the GluN2B subunit [271], and compared with the effects of Ro 25-6981, which is ~5000-fold selective for GluN2B over GluN2A subunit [272]. Our results showed that NVP-AAM077 and Ro 25-6981 possess antidepressant-like activity, and that neither of these compounds alone exhibit psychotomimetic-like activity [273]. However, the combination of NVP-AAM077 and Ro 25-6981 was sufficient to produce stereotypical behavior, which is associated to psychotic symptoms [273]. This would suggest that the blockade of either one of these subunits is sufficient to elicit an antidepressant-like action, but only when both subunits are blocked, psychotic-like effects would appear.

The GluN2D subunit has also been implicated in in some of the effects of the treatment with ketamine. Thus, it has been reported that the GluN2D subunit is crucial for the sustained antidepressant effects of (*R*)-ketamine. [274]. Further work from the same group of investigators has suggested that the GluN2D subunit plays also a role in cases of cognitive impairment induced by (*R*)-ketamine, whereas this subunit does not appear to be involved in cognitive impairment that is induced by (*R*,*S*)-ketamine or (*S*)-ketamine. [249,275].

Contrary to ketamine, the therapeutic mechanisms of conventional antidepressant drugs do not influence glutamatergic transmission in the brain neither under acute nor chronic regimen [276,277]. This lack of impact on glutamate, together with the fact that these drugs do not use mTOR as intracellular signal (see above), indicate that the antidepressant action of rapid-acting and conventional drugs follows different pathways.

## 6. Conclusions

In summary, ketamine has been used to model schizophrenia and to treat refractory depression. In both cases, the mechanism of action appears to be the same one, i.e., ketamine evokes cortical disinhibition by preferentially blocking NMDARs localized to PV interneurons [278,279]. Further work has suggested that blockade of GluN2B-containing NMDARs is responsible of the antidepressant-like effects of ketamine [279,280]. In fact, the excessive inhibition of hippocampal pyramidal neurons mediated by PV interneurons might contribute to depression-like behavior in an animal model [203] and GABA interneurons are the cellular trigger for ketamine’s rapid antidepressant actions. It is possible that psychosis is an immediate consequence of NMDA receptor blockade and reflects, in part, the ability of ketamine to induce glutamate release [104,281]. However, the antidepressant response emerges later, when glutamate released by ketamine would stimulate AMPA receptors [229,231,282]. Thus, both actions are mechanistically associated but temporally dissociated. It has been recently postulated that the mechanisms of rapid-acting antidepressant drugs converge on GluA1 receptors [236]. Thus, ketamine blocks the NMDAR channel, which leads to increases in extracellular glutamate and synapse number in the prefrontal cortex. This glutamate stimulates AMPA receptors which, through an activation of mTOR signaling pathway, induces a rapid synthesis of new proteins—in particular, BDNF and the GluA1 subunit—that are responsible for the rapid antidepressant effects.
